# A scoping review of the use of minimally important difference of EQ-5D utility index and EQ-VAS scores in health technology assessment

**DOI:** 10.1186/s12955-024-02272-9

**Published:** 2024-08-13

**Authors:** Caroline Shaw, Louise Longworth, Bryan Bennett, Louise McEntee-Richardson, James W. Shaw

**Affiliations:** 1 Putnam, Portland House, New Bridge Street West, Newcastle upon Tyne, NE1 8AP UK; 2 Worldwide Health Economics and Outcomes Research, Bristol-Myers Squibb Pharmaceuticals Limited, Uxbridge, UK; 3 Patient-Centred Outcomes, Jazz Pharmaceuticals, Oxford, UK; 4 Worldwide Health Economics and Outcomes Research, Bristol-Myers Squibb, Lawrenceville, NJ USA

**Keywords:** Minimally important difference, EQ-5D, Health technology assessment, Health-related quality of life

## Abstract

**Objectives:**

Estimates of minimally important differences (MID) can assist interpretation of data collected using patient-reported outcomes (PRO), but variability exists in the emphasis placed on MIDs in health technology assessment (HTA) guidelines. This study aimed to identify to what extent information on the MID of a commonly used PRO, the EQ-5D, is required and utilised by selected HTA agencies.

**Methods:**

Technology appraisal (TA) documents from HTA agencies in England, France, Germany, and the US between 2019 and 2021 were reviewed to identify documents which discussed MID of EQ-5D data as a clinical outcome assessment (COA) endpoint.

**Results:**

Of 151 TAs utilising EQ-5D as a COA endpoint, 58 (38%) discussed MID of EQ-5D data. Discussion of MID was most frequent in Germany, in 75% (*n* = 12/16) of Gemeinsamer Bundesausschuss (G-BA) and 44% (*n* = 34/78) of Institut für Qualität und Wirtschaftlichkeit im Gesundheitswesen, (IQWiG) TAs. MID was predominantly applied to the EQ-VAS (*n* = 50), most frequently using a threshold of > 7 or > 10 points (*n* = 13). G-BA and IQWiG frequently criticised MID analyses, particularly the sources of MID thresholds for the EQ-VAS, as they were perceived as being unsuitable for assessing the validity of MID.

**Conclusion:**

MID of the EQ-5D was not frequently discussed outside of Germany, and this did not appear to negatively impact decision-making of these HTA agencies. While MID thresholds were often applied to EQ-VAS data in German TAs, analyses were frequently rejected in benefit assessments due to concerns with their validity. Companies should pre-specify analyses of continuous data in statistical analysis plans to be considered for treatment benefit assessment in Germany.

## Introduction

Patient-reported outcome (PRO) measures, which assess patients’ perceived health-related quality of life (HRQoL) or health status, are increasingly included in clinical trials to support clinical efficacy and safety endpoints [1]. The EQ-5D is a generic PRO measure, comprising five health dimensions (mobility, self-care, usual activities, pain/discomfort, and anxiety/depression) and a visual analogue scale (VAS) [2], and is the most frequently preferred choice of instrument in health technology assessment (HTA) guidelines [3]. Two versions of the EQ-5D are available: the 3-Level EQ-5D (EQ-5D-3 L) with 3 severity levels for each dimension and the 5-Level EQ-5D (EQ-5D-5 L) with five severity levels [2]. In order to interpret PROs such as the EQ-5D, minimally important difference (MID) thresholds can be applied to determine whether change in scores translates into markers of clinical improvement, or via defining responders to treatments [4]. MID has been defined as “the smallest difference in score in the domain of interest that patients perceive as important, either beneficial or harmful, and which would lead the clinician to consider a change in the patient’s management” [5]. Terminology relating to MID can be confusing, with multiple terms that differ in definition, which have led to inconsistency in terminology used [6, 7]. Further, there are differences in methods for estimating MID and minimal important change (MIC), which vary in methodological robustness [7]. De Vet and Terwee (2010) highlight that while MIC and MID are frequently used interchangeably, the authors prefer the use of MIC instead of MID, in order to differentiate changes from differences [8].

Some guidance on the use of MID has been provided by regulatory agencies, such as the US Food and Drug Administration (FDA) and the European Medicines Agency (EMA) [9–11]. In order for both agencies to accept the clinical relevance of PRO data to support labeling claims, thresholds must be justified by the sponsor and defined a priori in the study protocol and statistical analysis plan [9–11]. Furthermore, while some leading HTA bodies such as the National Institute for Health and Care Excellence (NICE) and the Institute for Clinical and Economic Review (ICER) have not included information on the adoption of MID in their methods for health technology evaluations [12, 13], other agencies have incorporated it into their guidance. The Haute Autorité de Santé (HAS) recognises that MIDs can be used to overcome challenges of interpreting HRQoL data, however, data must be subject to rigorous methodology, with at least one clinical relevance threshold specified in study protocols, for assessment by the Commission de la Transparence [14]. More recently, Institut für Qualität und Wirtschaftlichkeit im Gesundheitswesen, (IQWiG) updated its General Methods in November 2020 and stated that responder analyses using an MID would be used for assessment, providing that the analyses were pre-specified in study protocols and the response criterion corresponds to at least 15% of the scale range of the PRO used [15].

MID threshold estimates can be derived through several approaches (e.g., anchor-based, distribution-based) and there is no consensus on an MID to use for the EQ-5D utility index and EQ-VAS [16]. Estimated thresholds can vary by patient population, clinical context, sociodemographic factors, and at the group level, depending on whether patients’ health status improves or deteriorates [6].

As part of a broader study to review the extent to which EQ-5D is used as a clinical outcome assessment endpoint in health technology assessment (HTA) decisions, regulatory labeling claims, and published literature [17], the objective of this study was to identify to what extent information on the MID of EQ-5D utility index and EQ-VAS scores is required and utilised by HTA agencies.

## Methods

### Literature search

Technology appraisal (TA) decision and supporting documents published over a two-year period (between 1 January 2019 and 15 January 2021) were identified and manually downloaded from the websites of five HTA bodies from France, Germany, UK and US: Haute Autorité de Santé (HAS, www.has-sante.fr/*)*, Institut für Qualität und Wirtschaftlichkeit im Gesundheitswesen, (IQWiG, www.iqwig.de/en/*)*, Gemeinsamer Bundesausschuss (G-BA, www.g-ba.de/*)*, National Institute for Health and Care Excellence (NICE, www.nice.org.uk/*)*, and Institute for Clinical and Economic Review (ICER, https://icer.org/*).* Searches were conducted on 15 January 2021, except for G-BA, where a search was conducted on 11 October 2021. Electronic keyword searching was then performed to identify documents including the following EQ-5D-related terms: EQ-5D, EQ5D, EQ-5D-3L, EQ-5D-5L, EuroQoL, EQ-VAS, and EQVAS.

### Study selection

All retrieved documents which included EQ-5D-related terminology were reviewed by one analyst, and 10% were reviewed by a second analyst. Records were included or excluded according to pre-specified eligibility criteria. Inclusion criteria for the broader HTA review included drug technologies intended for human use, and EQ-5D data (utility index and/or VAS) presented outside of the context of economic evaluation in guidance documents and supporting material. Appraisal documents which described non-drug technologies (e.g., medical devices, procedures, diagnostics, or digital applications), referred to EQ-5D data only in the context of economic evaluation, presented EQ-5D-Y data only, or those related to minor modifications of the marketing authorisation which did not provide additional data (e.g., ‘demande de renouvellement d’inscription’ or ‘application for renewal of registration’ reviews conducted by HAS) were excluded. Any disagreements between analysts were resolved through discussion until a consensus was reached.

### Data extraction and synthesis

Data from the included TAs were extracted by one analyst and quality checked by a second analyst. Data were extracted from guidance documents, and additional data (e.g., further analyses) were extracted from supporting documents (e.g., NICE committee papers or G-BA tragende gründe zum beschluss and zusammenfassende dokumentation), where available. As G-BA TAs were identified at a later date, abbreviated data extractions were performed for G-BA TAs, whereby only differences between data reported in linked G-BA and IQWiG documents (i.e., reporting the same product and indication) were extracted, to avoid duplication of data. Extracted data included drug assessment details, source, and type of EQ-5D data, whether MID was discussed, the level of MID applied and its source, and HTA agency comments about the application of MID. Where outcome data were missing, they were extracted as “not reported”. Data were presented descriptively, using a combination of narrative synthesis and summary tables to present frequencies of MID use. MID values were grouped into pre-specified thresholds, based on MID estimates for the EQ-5D utility index (UK scores) and EQ-VAS for cancer [18]. No statistical comparative analyses were performed. Differences between data reported in linked IQWiG and G-BA documents for German HTA submissions were also presented descriptively.

## Results

### Literature search

A detailed breakdown of the flow of studies in the HTA review has been described previously [17]. In summary, 1329 HTA decision and supporting documents from 1072 technology appraisals were identified in the literature search. After screening for eligibility, 298 documents from 195 TAs met the inclusion criteria (G-BA *n* = 60, HAS *n* = 11, ICER *n* = 3, IQWiG *n* = 78, NICE *n* = 43). However, only 16 of the 60 G-BA TAs meeting the inclusion criteria provided additional EQ-5D data to linked IQWiG TAs and were extracted. Therefore, 151 TAs were considered for MID data.

### Discussion of minimally important difference

Of the 151 TAs included in the HTA review which provided unique data, only 38% (*n* = 58/151) discussed the MID of EQ-5D data (Table 1). German appraisals most frequently mentioned the MID, in 75% (*n* = 12/16) of G-BA TAs and 44% (*n* = 34/78) of IQWiG TAs. ICER mentioned the MID in 33% (*n* = 1/3) and NICE in 23% (*n* = 10/43) of appraisals. Discussion of MID occurred less often in French appraisals (*n* = 1, 9%). Cancer was the most frequently addressed disease, in 91% of appraisals mentioning MID (*n* = 53/58). All G-BA appraisals were on cancer (*n* = 12, 100%), all HAS appraisals on blood or immune disease (*n* = 1, 100%) and all ICER on digestive disease (*n* = 1, 100%). Among IQWiG appraisals discussing MID, 94% (*n* = 32) were on cancer and 6% on musculoskeletal issues (*n* = 2), with 90% of NICE appraisals on cancer (*n* = 9) and 10% on musculoskeletal issues (*n* = 1).

While terminology for MID was variable (minimal(ly) important difference, minimal clinically important difference, clinically meaningful, clinically meaningful difference, clinically meaningful change, clinically meaningful improvement, clinically relevant improvement, clinically relevant deterioration), the term ‘minimal(ly) important difference’ was most frequently reported, in 72% of TAs mentioning MID (*n* = 42/58). However, limited explanation of the methodology utilised made it difficult to assess whether the correct terms were employed. In contrast to Terwee et al’s 2021 definition of MIC as longitudinal and MID as cross-sectional [7], the G-BA considered MID as longitudinal [19–27].

**Table 1:** Discussion of minimally important difference, stratified by HTA agency

	Discussion of MID		
**HTA agency**	**Yes** **n (%)**	**No** **n (%)**	**Total** **n (%)**
G-BA	12 (75)	4 (25)	16 (100)
HAS	1 (9)	10 (91)	11 (100)
ICER	1 (33)	2 (67)	3 (100)
IQWiG	34 (44)	44 (56)	78 (100)
NICE	10 (23)	33 (77)	43 (100)
Total	58 (38)	93 (62)	151 (100)

Abbreviations: G-BA, Gemeinsame Bundesausschuss; HAS, Haute Autorité de Santé; HTA, health technology assessment; ICER, Institute for Clinical and Economic Review; IQWiG, Institut für Qualität und Wirtschaftlichkeit im Gesundheitswesen; MID, minimally important difference; NICE, National Institute for Health and Care Excellence

Of those which mentioned the MID, a greater proportion discussed the MID for the EQ-VAS (86%) than the EQ-5D utility index (5%), or the utility index and EQ-VAS in combination (5%; see Table 2). Forty six of the 53 (87%) appraisals which discussed the MID for the EQ-VAS were German.

**Table 2:** Discussion of minimally important difference, stratified by EQ-5D measure and HTA agency

	EQ-5D measure				
**HTA agency**	**Utility index only** **n (%)**	**EQ-VAS only** **n (%)**	**Utility index + EQ-VAS** **n (%)**	**NR** **n (%)**	**Total** **n (%)**
G-BA	0 (0)	12 (100)	0 (0)	0 (0)	12 (100)
HAS	0 (0)	0 (0)	1 (100)	0 (0)	1 (100)
ICER	0 (0)	1 (100)	0 (0)	0 (0)	1 (100)
IQWiG	0 (0)	34 (100)	0 (0)	0 (0)	34 (100)
NICE	3 (30)	3 (30)	2 (20)	2 (20)	10 (100)
Total	3 (5)	50 (86)	3 (5)	2 (4)	58 (100)

Abbreviations: G-BA, Gemeinsame Bundesausschuss; HAS, Haute Autorité de Santé; HTA, health technology assessment; ICER, Institute for Clinical and Economic Review; IQWiG, Institut für Qualität und Wirtschaftlichkeit im Gesundheitswesen; NICE, National Institute for Health and Care Excellence; NR, not reported; VAS, visual analogue scale

### EQ-5D MID thresholds reported

Reported MID thresholds stratified by HTA agency are summarised in Table 3. Of the 58 appraisals which mentioned MID, 50 (86%) reported the threshold utilised (thresholds were reported for both the EQ-5D utility index and EQ-VAS in 1 NICE [28] and in 1 HAS TA [29]). Only NICE and HAS reported using MID thresholds for the EQ-5D utility index in 5 TAs [28–32] and none of these were utilised more than once. Of the appraisals which specified the MID threshold used for the EQ-VAS, 100% reported MID thresholds of ≥ 7 points (*n* = 47). A threshold of > 7 or > 10 points was most frequently used for EQ-VAS data (28%), including in 2 G-BA [23, 24] and 11 IQWiG TAs [33–43].

**Table 3:** EQ-5D MID thresholds reported, stratified by HTA agency

	HTA agency					
**Level of MID applied**	**G-BA** **n (%)**	**HAS** **n (%)**	**ICER** **n (%)**	**IQWiG** **n (%)**	**NICE** **n (%)**	**Total** **n (%)**
**EQ-5D utility index**						
< 0.08	0 (0)	0 (0)	0 (0)	0 (0)	1 (100)	1 (100)
≥ 0.08	0 (0)	0 (0)	0 (0)	0 (0)	3 (100)	3 (100)
Other ^a^	0 (0)	1 (100)	0 (0)	0 (0)	0 (0)	1 (100)
Total	0 (0)	1 (20)	0 (0)	0 (0)	4 (80)	5 (100)
**EQ-VAS**						
< 7	0 (0)	0 (0)	0 (0)	0 (0)	0 (0)	0 (0)
≥ 7	11 (23)	1 (2)	1 (2)	30 (64)	4 (9)	47 (100)
Total	11 (23)	1 (2)	1 (2)	30 (64)	4 (9)	47 (100)

Abbreviations: G-BA, Gemeinsame Bundesausschuss; HAS, Haute Autorité de Santé; ICER, Institute for Clinical and Economic Review; IQWiG, Institut für Qualität und Wirtschaftlichkeit im Gesundheitswesen; MID, minimally important difference; NICE, National Institute for Health and Care Excellence; VAS, visual analogue scale

^a^ Other values which do not fit into specified thresholds: >0.07 (HAS)

### Source of MID thresholds

Of the 58 appraisals which mentioned MID, 40 (69%) reported the source of the thresholds used (1 NICE TA reported the same source for both the EQ-5D utility index and EQ-VAS [28]). As shown in Table 4, only 2 TAs published by NICE reported the source of MID thresholds utilised for the EQ-5D utility index [28, 30], which equally referenced Walters & Brazier 2005 [44] and Delaloge et al. 2019 [45]. For EQ-VAS data, Pickard et al. 2007 [18] was most frequently reported (90%), including in 11 G-BA [19–27, 46, 47] and 24 IQWiG TAs [33, 34, 36, 39, 41–43, 48–64].

**Table 4:** Source of MID thresholds for EQ-5D utility index and EQ-VAS

Reference	EQ-5D utility index *n* (%)	EQ-VAS *n* (%)
Pickard et al. (2007) [18]	0 (0)	35 (90)
Perl et al. (2017) [65]	0 (0)	1 (3)
Coteur et al. (2009) [66]	0 (0)	1 (3)
Hurst et al. (1997) [67]	0 (0)	1 (3)
Delaloge et al. (2019) [45]	1 (50)	1 (3)
Walters & Brazier (2005) [44]	1 (50)	0 (0)
Total	2 (100)	39 (100)

Of the TAs which reported the source of MID (*n* = 40), 38 (95%) were for cancer, one ICER TA was for digestive tract conditions, and one IQWiG TA was for musculoskeletal conditions. Almost all applied MID thresholds to patient populations with the same indication as the source (95%, *n* = 38). The exceptions to this include 1 IQWiG TA in cancer [68] utilising rheumatoid arthritis-specific MID for the EQ-VAS by Hurst et al. (1997) [67], and 1 IQWIG TA for osteoporosis [62] utilising cancer-specific MID for the EQ-VAS by Pickard et al. (2007) [18].

### Differences in MID between G-BA and IQWiG appraisals of the same product

When MIDs were compared between G-BA and IQWiG appraisals of the same product and indication (linked appraisals), 4 (25%) G-BA appraisals which presented additional EQ-5D data reported different MID usage [21, 22, 26, 47] (Table 5). In all cases, the MID threshold was reported in G-BA and not in IQWiG TAs. For 31% of G-BA TAs, MID thresholds were not reported in either one or both of the linked IQWiG and G-BA documents.

**Table 5:** Differences between reported MID in linked G-BA and IQWiG appraisals

Was MID the same between G-BA and IQWiG TAs?	Total *n* (%)
Yes, same	7 (44)
No, different	4 (25)
NR/NA	5 (31)
Total	16 (100)

Abbreviations: G-BA, Gemeinsame Bundesausschuss; IQWiG, Institut für Qualität und Wirtschaftlichkeit im Gesundheitswesen; MID, minimally important difference; NA, not applicable; NR, not reported; TA, technology appraisal

### Acceptability of EQ-5D MID data

In 34 appraisals, HTA agency comments were provided about the acceptability of the MID source and/or thresholds applied by the submitting companies, almost all of which were from Germany (G-BA *n* = 12, IQWiG *n* = 19, NICE *n* = 3). In 2 NICE TAs [30, 69], it was noted that there was a lack of clarity about the MID thresholds applied, and results should be interpreted cautiously due to small patient sample sizes in another [32]. In a fourth NICE TA, the Evidence Review Group stated it was “satisfied that the company’s approach to analysing patient-reported outcomes was pre-specified” (including applying an MID of ≥ 0.08 to the EQ-5D-5 L utility index) and that the approach was appropriate [31].

However, German HTA agencies were more critical of MID data analyses, particularly in reference to a lack of pre-specification of the MIDs utilised [37, 68, 70] and their source. In 13 TAs, IQWiG criticised the use of Pickard et al. 2007 [18] as the source of MID thresholds for the EQ-VAS, as it was perceived as being unsuitable for assessing the validity of MID [33, 34, 36, 39, 41, 42, 48, 49, 56–58, 62, 63]. Consequently, MID analyses were excluded from the benefit assessment. Similarly, in the assessment of daratumumab (Darzalex, Janssen-Cilag International NV) [68], analyses of EQ-VAS data based on MIDs estimated by Hurst et al. 1997 [67] were also considered to be inappropriate and excluded from the benefit assessment, as it was noted that a MID for the EQ-VAS was not examined in Hurst et al. 1997 [67].

The G-BA echoed the opinion of IQWiG that the MID from Pickard et al. 2007 [18] was unsuitable, as the MID was not derived from a longitudinal study [19–27]. Furthermore, the G-BA stated that the Eastern Cooperative Oncology Group Performance Scale (ECOG-PS) and Functional Assessment of Cancer Therapy - General (FACT-G) total score anchors used in the study were also not considered by IQWiG to be suitable for deriving a MID, however the reasoning for this was not provided [19–21, 26]. In several cases, IQWiG utilised continuous analyses of EQ-VAS data (e.g., standardised mean differences [a summary statistic where standard deviations are used to standardise results of studies to a single, weighted scale [71]] in EQ-VAS score, expressed as Hedges’ g [an effect size measure representing the standardised difference between means [72]]) instead of responder analyses (the proportion of patients achieving a pre-defined level of improvement [73]) based on a MID [19–24, 26, 27, 70]. Nevertheless, the G-BA differed from IQWiG and considered responder analyses using the EQ-VAS in its decision making, citing that responder analyses based on a MID for clinical evaluation of effects have advantages over analyses of standardised mean value differences [19–26, 47, 70].

## Discussion

In the context of HTA decision making, this study highlighted that estimates of MID are infrequently used to analyse and interpret EQ-5D data outside of Germany. Overall, 38% of included records (*n* = 58/151) discussed MID of the EQ-5D in some context, 79% (*n* = 46/58) of which were from Germany. Considering we found in the broader HTA review that 100% of IQWiG and 94% of G-BA TAs reporting EQ-5D data for COA were for the EQ-VAS only [17], it was perhaps unsurprising that 86% of all TAs and 100% of German TAs mentioning MID were for the EQ-VAS. Due to the small proportion of TAs discussing MID for the EQ-5D utility index (*n* = 5, 9%), limited conclusions can be drawn from the data. Thresholds were reported in 1 HAS and 4 NICE TAs and sources were provided for 2 TAs, but none of which were duplicated. However, NICE did note in 1 TA that the approach used to analyse EQ-5D utility index data was appropriate [31].

Pickard et al., 2007 [18] was the most frequently cited source of MID, in 88% (*n* = 35/40) of TAs which reported the source and was exclusively used for the EQ-VAS in German submissions. In this reference, Pickard et al. estimated cancer-specific MIDs for EQ-VAS scores ranging from 7 to 10, when MIDs were averaged across the anchor-based categories derived using FACT-G quintiles. In our review, we found 10 different variations in MID around the 7 and/or 10-point threshold from TAs quoting this source, with scores greater than 7 or greater than 10 points as the most frequently reported MID. We also found that of the TAs which reported the source of MID (*n* = 40), almost all applied thresholds to patient populations with the same indication as the source (95%, *n* = 38).

While HAS recognises the benefits of using MIDs in its guidance [14], currently, there are no recommended MID thresholds for NICE, HAS, or ICER. However, in November 2020, IQWiG introduced a value of at least 15% of the scale range of the generic or disease-specific instrument used, which was derived from the findings of a systematic literature review of MIDs in 8 therapeutic areas [15]. As there is no universal MID estimate to use for each PRO, and MIDs can be highly variable, IQWiG adopted this approach to ensure that suitable response thresholds are used in responder analyses for benefit assessments and to minimise selective outcome reporting, which could arise by selecting one of many available MIDs. As the EQ-VAS is predominantly used in Germany, and the scale ranges from 0 to 100 points, this criterion equates to an improvement in responses of 15 points or above. In this review, we found that no TAs reported using thresholds starting at or above 15 points for the EQ-VAS. The highest threshold utilised was 12 points in a NICE TA of gilteritinib for treating relapsed or refractory acute myeloid leukaemia [74]. Furthermore, despite the availability of MID estimates for the EQ-VAS in disease areas such as chronic obstructive pulmonary disease, oncology, osteoarthritis, and Crohn’s disease [18, 66, 75–78], we were unable to identify MID estimates that meet IQWiG’s recommendations. It is therefore possible that this new MID requirement could be unrealistically large for the EQ-VAS and could result in fewer products gaining added value benefit based on PRO data. Further research is required to identify whether a 15% improvement in the EQ-VAS is a minimally meaningful change as perceived by patients.

Discussion of the acceptability of EQ-5D MID data varied between HTA agencies. There was no mention of it in the included appraisals by HAS and ICER. Four NICE TAs included agency comments related to the MID of EQ-5D data, one of which was favourable, and all except 1 drug were recommended. Given that these HTA agencies have not published recommendations on MID thresholds to use (or even discussed MID in guidance documents), the low frequency of TAs discussing MID does not appear to have negatively impacted the final decision making on drug technologies by HAS, ICER, or NICE.

Conversely, the acceptability of EQ-5D MID data was frequently discussed in German TAs, including 12 G-BA and 19 IQWiG TAs. Key criticisms referred to a lack of pre-specifying MID analyses in study protocols and the validity of the thresholds used. Principally, IQWiG did not utilise EQ-VAS responder analyses in submissions citing Pickard et al. 2007 [18] as this source was not deemed suitable to demonstrate validity of the EQ-5D MID. In agreement, the G-BA further elaborated that the main concern related to the cross-sectional design of the study underpinning Pickard et al.’s MID analyses. Concerns were also expressed about the choice of anchors. In these cases, the G-BA noted that IQWiG utilised continuous analyses of EQ-VAS (e.g., standardised mean differences in EQ-VAS score, expressed as Hedges’ g) instead of responder analyses based on a MID. However, contrary to these criticisms, the G-BA still considered responder analyses in its decision making, due to preferring process consistency and recognising the advantages of using responder analyses based on a MID compared with analyses of standardised mean value differences. Since the searches were performed in this literature review, the G-BA has adopted the mandatory requirement to use the 15% threshold as suggested by IQWiG [79] to define the MID threshold used in responder analyses. Therefore, in future, we anticipate the exclusion of EQ-VAS responder analyses from benefit assessments in a greater number of TAs where chosen MIDs do not meet the 15% threshold. Pharmaceutical companies should consider PRO requirements that are relevant for HTA decision-making when designing clinical trials. Until MIDs meeting a 15% threshold for the EQ-VAS are available, companies should include the pre-specification of analyses of continuous data (i.e., standardised mean differences expressed as Hedges’ g) in statistical analysis plans in order to be considered for treatment benefit assessment in Germany.

### Strengths, limitations, and scope for further work

This study incorporated appraisals from multiple HTA agencies from the same time period, which allowed for direct comparison of EQ-5D MID data across different markets. Five agencies were chosen for review, as they are leading global HTA bodies which release publicly available and transparent documents for each technology. However, they may not necessarily reflect the use of EQ-5D amongst other agencies. Further investigation across additional HTA agencies could help expand the context of the results detailed here. It is also important to note that searching of G-BA documents was added at a later date, therefore data are not presented in the same way as for other agencies. This is because abbreviated extractions were performed which involved focus on additional data above what were reported in the linked IQWiG documents, so as not to introduce duplicated data.

Another limitation surrounds the chosen two-year timeframe in the search strategy. As the searches were not limited by disease area or drug technology, there was a large volume of articles to be screened. While this approach allowed exploration of trends between HTA agencies as part of the broader literature review, there were relatively low numbers of included TAs which mentioned MID for some HTA agencies. Furthermore, searches were conducted two months after IQWiG updated its guidance on the use of MID for analysing PRO data. Further research is warranted to identify longitudinal trends in MID usage, and whether these guidelines have affected the proportion of drug assessments with accepted PROs and benefit ratings affected by PROs, since coming into effect.

## Conclusions

The MIDs of EQ-5D outcomes were not frequently discussed in HTA dossiers outside of Germany, and this did not appear to negatively impact the decision-making of HTA agencies. While MID thresholds were often applied to EQ-VAS data in German TAs, these analyses were frequently rejected from benefit assessments, due to concerns with the validity of their source. Furthermore, although most thresholds for the EQ-VAS were greater than 7 or 10 points, no thresholds started at or above IQWiG’s recommended threshold of 15 points. Companies should carefully consider utilising appropriate MID thresholds according to HTA agency requirements, to demonstrate product value during clinical trial design. Specifically for Germany, until MIDs meeting a 15% threshold for the EQ-VAS are available, study sponsors should include the pre-specification of analyses of continuous data (i.e., standardised mean differences expressed as Hedges’ g) in statistical analysis plans to be considered for treatment benefit assessment.

## Data Availability

No datasets were generated or analysed during the current study.
